# Total Body Surface Area Adjusted Daily Diagnostic Blood Loss May Be Higher in Minor Burns—Are Our Patients the Victims of Daily Routine?

**DOI:** 10.3390/ebj5020016

**Published:** 2024-06-06

**Authors:** Christian Smolle, Anna Alexandra Elisabeth Persson, Caroline Lind, Fredrik Huss

**Affiliations:** 1 Burn Center, Department of Plastic and Maxillofacial Surgery, Uppsala University Hospital, 751 85 Uppsala, Sweden; christian.smolle@medunigraz.at (C.S.); caroline.lind@regionkalmar.se (C.L.); fredrik.huss@akademiska.se (F.H.); 2 Division of Plastic, Aesthetic and Reconstructive Surgery, Department of Surgery, Medical University of Graz, 8010 Graz, Austria; 3 Research Unit Safety in Health, Division of Plastic, Aesthetic and Reconstructive Surgery, Medical University of Graz, 8010 Graz, Austria; 4 Department of Surgical Sciences, Plastic Surgery, Uppsala University, 751 85 Uppsala, Sweden

**Keywords:** diagnostic blood loss, burn injury, hospital-acquired anaemia, transfusion, total body surface area

## Abstract

Burns are common and devastating injuries, often necessitating intensive care treatment and long-term hospitalisation, making burn patients susceptible to hospital-acquired anaemia and blood transfusion. The purpose of this study was to assess diagnostic blood loss in burn patients at the burn intensive care unit (BICU) at Uppsala University Hospital between 1 September 2016 and 30 June 2019. Medical records were screened; age, gender, mechanism, % total body surface area (TBSA), Baux score, length of stay, days on the respirator, days of continuous renal replacement therapy, number of operations, and number of blood tests per patient were assessed. Volume per blood test was estimated as the volume needed for the specific test tube. A total of 166 patients were included in the study. The mean TBSA was 18.0% ± 20, and the mean length of stay was 17.0 ± 41 days. Median diagnostic blood loss was 13.1 mL/day/patient (IQR 7.0, 23.9) and correlated positively with burn extent, Baux score, and mortality. Daily diagnostic blood loss/%TBSA/patient was 1.2 mL (IQR 0.7, 2.3). Transfusion of blood products occurred in 73/166 patients (44%). In conclusion, diagnostic blood loss is greatly influenced by TBSA extent. The diagnostic blood loss can reach significant levels and may affect the transfusion rate.

## 1. Introduction

Burns are common and severe injuries, often necessitating intensive care treatment and long hospitalisation [1,2,3]. Throughout hospitalisation, burn patients are susceptible to hospital-acquired anaemia (HAA) for several reasons: The burn injury itself often causes impaired hemopoiesis [4], whereas repeated surgeries and dressing changes often result in significant blood loss [5,6,7,8]. Further reasons for HAA in burn patients include episodes of systemic inflammatory response, comorbidities, and coagulopathies associated with the insult of the injury. On top of that, patients undergoing intensive care are subjected to extensive diagnostic blood sampling, which can lead to iatrogenic anaemia [9]. Anaemia itself has been linked to inferior outcomes, transfusion risks, and a significant impact on patient morbidity and mortality [10].

Consequently, burn patients often experience a drop in their blood count, and it is not unusual for them to need a blood transfusion. It is therefore particularly important to reflect on whether all blood samples are essential for the patient’s therapy or if some can be spared. Our hypothesis was that diagnostic blood loss in burn patients can reach significant levels and may have a clinical impact. This said, the risks of iatrogenic anaemia must be weighed against the consequences of absent information from repeated blood testing.

A few studies have been conducted on diagnostic blood loss in ICU patients, but to our knowledge, there are not many studies on diagnostic blood loss in extensive burn patients. In a study by Yao R et al. in 2019, blood loss due to phlebotomy for diagnostic purposes was advocated to be a relevant factor for the development of HAA in major burns [11].

The aim of this study was to quantify diagnostic blood loss (DBL) in patients with minor and major burn injuries and to assess factors associated with increased DBL and transfusion of blood products in both groups.

## 2. Materials and Methods

### 2.1. Patients and Collected Clinical Data 

The medical records of all patients admitted to the Burn Intensive Care Unit (BICU) of the burn centre in Uppsala in Sweden between 1 September 2016 and 30 June 2019 were retrospectively screened. Basic medical data were acquired from the internal burns documentation system and included patient age, gender, % burnt total body surface area (%TBSA), Baux score, burn cause, days on the respirator, days of continuous renal replacement therapy (CRRT), number of operations, length of stay (LOS), and outcome. Patients were excluded if they were under the age of 18, if they had been transferred to the BICU > 48 h after the injury, if medical records were incomplete, if no blood tests had been conducted, or if they had been admitted to the BICU for a reason other than a burn (e.g., necrotising fasciitis, toxic epidermal necrolysis). Depending on bed capacity in the normal ward, burn patients not requiring intensive care were frequently treated at the BICU; for this reason, minor burns requiring hospitalisation were also included in the study.

### 2.2. Estimation of Diagnostic Blood Loss 

The number and type of blood samples taken per patient were acquired from the hospital documentation system. Diagnostic blood loss (DBL) resulting from each test was estimated according to the recommended filling level of the respective blood vials at Uppsala University Hospital. The rationale for this approach was motivated by the fact that all BICU patients had their samples taken either from central venous lines or arterial lines, which made maximum filling likely. Accordingly, the estimated DBL was 4 mL for complete blood count (CBC), 5 mL for serum chemistry laboratory (SCL), 3.5 mL for coagulation testing (CT), 2 mL for blood gas analysis (BGA), 2 mL for CRRT citrate coagulation testing (CCCT), and 10 mL for each blood culture (BC) taken. Particular blood tests (i.e., hormone profile, drug levels) were taken infrequently and therefore summarised as “other tests” and were assigned 5 mL each. DBL per day was calculated by division of DBL by LOS, and DBL per percent TBSA was calculated by division of DBL by %TBSA. It has to be pointed out that these values only apply to the daily clinical routine performed at the Uppsala burn centre. It is likely that these estimates differ grossly from those of other centres depending on the laboratory equipment used.

According to observations of 118 blood sample draws from central venous or arterial lines in the BICU, the blood discarded (to flush the lines) per draw amounted to 2.5 mL per draw on average. Since it was not possible to quantify the number of discarded blood vials per patient retrospectively (based on the assumption that, most often, more than one sample had been taken per draw), these data were not included in further analyses to avoid gross overestimation of diagnostic blood loss.

### 2.3. Collection of Transfusion Data

Data on the number and type of blood product transfusions per patient were taken from the hospital documentation system as well. Blood products included erythrocyte concentrates (ECs, 255 mL each), fresh frozen plasma (FFP, 290 mL each), and thrombocyte concentrates (TCs, 200 mL each). ECs were given routinely at haemoglobin levels < 7 g/dL (according to Palmieri et al. [12]) or perioperatively when acute blood loss was anticipated or evident. Indication for FFP was major or critical bleeding, in line with mass transfusion protocol; EC/FFP/TC in the proportions 4:4:1. Furthermore, TCs were given if thrombocyte counts fell below 50 × 10^9^/L.

### 2.4. Statistical Analysis

Statistical analysis was carried out using SPSS Statistics 24.0 (IBM Inc., Armonk, NY, USA). Normally distributed continuous variables were provided as means and standard deviation (SD); parameters that did not follow a normal distribution were provided as the median and interquartile range (IQR). Univariate correlation analysis was conducted using the chi2 test for nominal variables. For normally distributed continuous parameters, Student’s *t*-test was used, while not-normally distributed continuous as well as discrete parameters were analysed using the Mann–Whitney U-test. Spearman’s correlation analysis was used to analyse correlations between two continuous parameters. Stepwise backwards linear regression analysis was applied to evaluate the relative influence of different parameters on DBL, and Pearson correlation was used to measure the strength of such associations. In all cases, a *p*-value of <0.05 was considered as the statistical level of significance.

## 3. Results

### 3.1. Patient Demographics and Clinical Data

A total number of 253 patients received care at the burn centre in Uppsala between 1 September 2016 and 30 June 2019. Of these, 77 patients were aged < 18 years and, therefore, excluded from the study along with one patient who did not have any blood tests, eight patients who were admitted because of toxic epidermal necrolysis, and one patient who was admitted due to frostbite. The study population hereafter consisted of 166 adult burn patients whose demographics are presented in Table 1.

**Figure 1:**
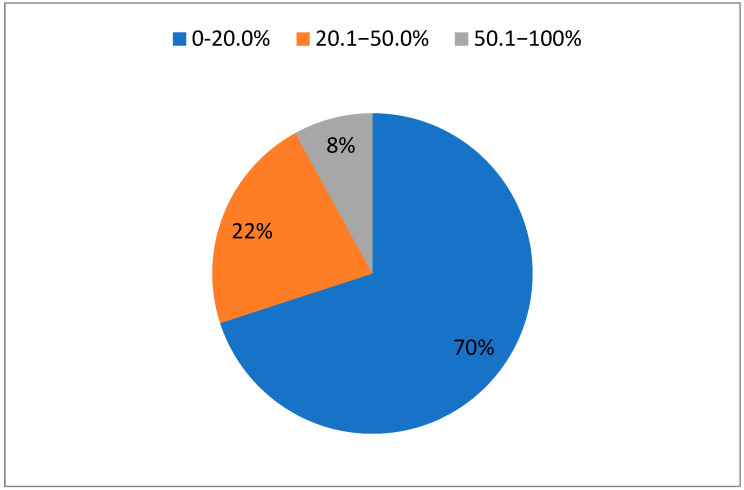
Distribution of burn injury extent according to %TBSA.

A majority of the burn patients were men (66.3%). The female patients were significantly older than the male patients (independent *t*-test, *p* < 0.05) with a mean age of 57.6 years compared to 51.0 years. The overall predominant mechanisms behind the treated injuries were flame (76.5%), scald (13.3%), and electric (7.2%). There was, however, a significant difference in the distribution of burn mechanisms between genders (chi2 test, *p* < 0.05), where scalds were significantly more common in women (chi2 test, *p* < 0.001). The mean %TBSA for the whole group was 18.0% (±20.1), and less than a third of the patients had a burn extent estimated to be more than 20% of the total body surface area, i.e., classified as a major burn (see Figure 1). There was a vast variation in how long the patients stayed at the burn centre, from 1 to 410 days with an average of 17.1 days (±40.7). In this patient material, the mean LOS/%TBSA was 1.9 days (±4.0) in total and 1.5 days (±2.4) if patients with electrical injuries were excluded.

In patients requiring surgery (74.7%), a mean of 1.9 procedures were performed. For those who required mechanical ventilation (25.3%), the median respirator treatment was 4 days. Six (3.6%) patients needed dialysis during the course of their treatment, and the median number of days with CRRT was 3.5. The calculation of the mortality index Baux score resulted in an average of 71.2 (±27.0). In this study population, 20 out of 166 patients (12%) died during their burn care and had a median Baux score of 114.8. Non-survivors also had significantly higher %TBSA than the survivors (Mann–Whitney U-test, *p* < 0.001) with a mean TBSA of 48.4% (±26) vs. 14.5% (±15). There was no significant correlation between mortality and age (Student’s *t*-test, *p* > 0.05) or between mortality and gender (chi2 test, *p* > 0.05). Details are presented in Table 1.

### 3.2. Diagnostic Blood Loss

As for the amount of blood drawn from each patient, the volume was calculated according to Table 2. The total number and volume of each category of blood analyses, sampled during the studied period, are presented in Table 3. The overall most frequent blood sample analysed was blood gas followed by complete blood count (CBC) and chemistry. On the other hand, concentrating on how many patients were subjected to diagnostic testing for each blood test category, the most ordered test was CBC (95.8% of patients), followed by chemistry (95.2% of patients) and coagulation (95.2% of patients). Focusing on the volume, on average, patients lost the biggest quantity of blood via blood culture testing (mean 176.4 mL), followed by blood gas analysis (mean 140.6 mL/patient). Regarding the volume of phlebotomy waste, the observations at the department yielded 118 documented sampling occasions with a mean volume of 2.5 mL per sampling occasion (range: 0.2–11.0).

The median total DBL as well as daily DBL per patient was analysed in relation to %TBSA (divided into groups of up to 20%, 20.1–50%, and 50.1–100%) and length of stay (see Table 4). Analysis of the entire collective irrespective of TBSA groups showed a median volume of 13.1 mL DBL per day per patient (IQR: 7.0, 23.9) along with a median volume of 10.8 mL DBL per %TBSA (IQR: 4.6, 27.8). There was a statistically significant correlation between daily DBL and %TBSA (Pearson correlation, *p* < 0.001), Baux score (Pearson correlation, *p* < 0.001), and mortality (independent *t*-test, *p* < 0.001), respectively. There was, however, no statistically significant correlation with length of stay.

### 3.3. Daily DBL Adapted for %TBSA

**Figure 2:**
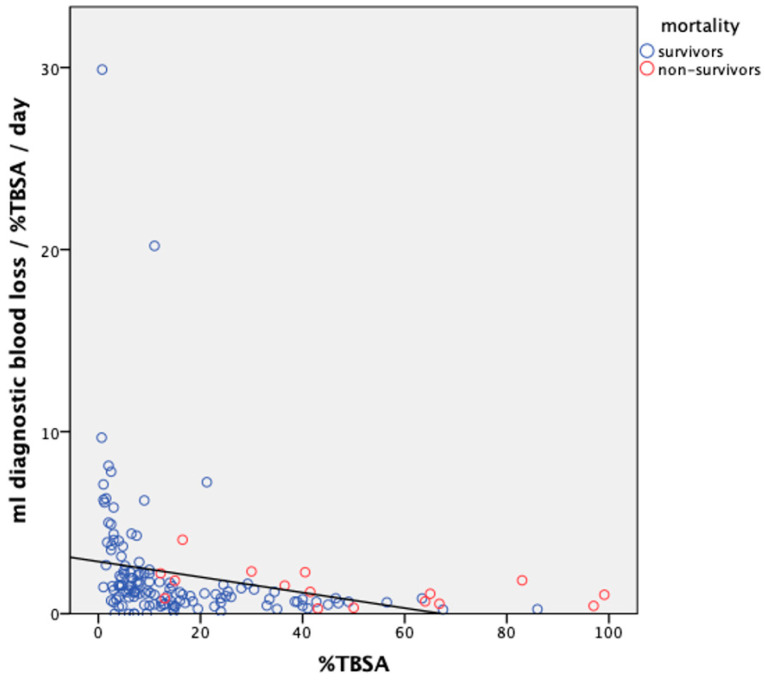
mL diagnostic blood loss per %TBSA per day slightly decreasing with increasing burn size (%TBSA, electrical burns not included). In the graph, each circle corresponds to one patient (blue = survivors, red = non-survivors). In black is the linear regression line.

Additionally, a calculation of DBL per %TBSA per day displayed that the burn patients in the study population had a median DBL of 1.2 mL/%TBSA/day (IQR: 0.7, 2.3). A linear logistic regression analysis was performed including the parameters age, gender, mechanical ventilation, dialysis, electrical injury, number of operations, length of stay, and %TBSA, weighted for “ml diagnostic blood loss per %TBSA per day” (mL DBL/%TBSA/d). In this model, the presence of an electrical burn was the sole parameter that correlated positively with higher ml DBL/%TBSA/d (β = 65.959, *p* < 0.001), whereas none of the other parameters had a significant impact. When patients with electrical burns were excluded from the analysis, %TBSA remained the only significant predictor, showing a tendency to correlate negatively with ml DBL/%TBSA/d (β = −0.049, *p* = 0.006, see Figure 2).

For further analysis, the patient collective was divided into two groups according to the median DBL/d: high daily DBL (hDBL/d ≥ 13.1 mL/d) and low daily DBL (lDBL/d < 13.1 mL/d). According to this analysis, patients in the hDBL/d group had significantly higher mean %TBSA (26.2 vs. 10.3, independent *t*-test, *p* < 0.001), higher Baux scores (mean 79.0 vs. 64.0, independent *t*-test, *p* < 0.001), longer mean LOS (24.5 vs. 10.1 days, independent *t*-test, *p* = 0.029), and higher mortality (17.5% vs. 7.0%, chi2 test, *p* = 0.037). Furthermore, hDBL/d was more likely after flame burns (83.8% vs. 69.8%, chi2 test, *p* = 0.034), associated with a higher number of operations (mean 2.9 vs. 1.1, independent *t*-test, *p* < 0.001), and more common in patients needing mechanical ventilation (42.5% vs. 9.3%, chi2 test, *p* < 0.001). On average, patients with high DBL/d spent more days on the respirator (mean 2.6 vs. 0.2, independent *t*-test, *p* < 0.001; for detailed results, see Table 5).

### 3.4. Transfusion Data

A total of 73 out of 166 patients (44%) received at least one transfusion of either fresh frozen plasma, erythrocyte concentrate, or thrombocyte concentrate during their stay at the burn centre, with erythrocyte concentrate being the most common blood product given. Median transfusion amounts per patient were 1785.0 (IQR 2805.0) mL for ECs, 1160.0 (IQR 2030.0) mL for FFP, and 400.0 (IQR 550.0) for TCs. Patients with major burns received >80% of all ECs administered and >90%, and >95% of all FFP and TCs, respectively. Furthermore, patients with major burns were significantly more likely to receive transfusions of any kind, while median transfusion amounts per patient were significantly higher for ECs and FFP but not for TCs.

## 4. Discussion

In this study, we retrospectively estimated DBL and the amount of blood transfusions in adult patients treated for burn injuries at the burn centre of Uppsala University Hospital. The results showed that these patients had a diagnostic blood loss of approximately 1.2 mL per %TBSA per day. Consequently, a patient with a burn injury extent of 40%TBSA, who is thereby anticipated to have an LOS of 40 days, can be expected to have an estimated blood loss of nearly 2 L (40 × 40 × 1.2 = 1920 mL) for diagnostic purposes alone. The amount of DBL per %TBSA per day correlated solely with %TBSA when electrical burns, which were considered as severe injuries despite low %TBSA, were excluded from the analysis. Apart from burn extent and length of stay, it was also found that the amount of daily DBL was positively correlated to Baux score and mortality.

The principal blood tests responsible for the DBL were found to be blood cultures (highest volume), blood gases (most frequently ordered), and complete blood count (performed in the highest number of patients). An approach with the aim of minimizing DBL could thus be to review the indications for sampling blood cultures at the burn centre. Current practice is the liberal drawing of blood cultures for every temperature above 39 °C. This routine has been established, as clinical practice has shown that conventional inflammatory biomarkers such as c-reactive protein and leucocyte counts do not allow for discrimination between systemic inflammatory response syndrome or actual sepsis in severe burns, especially not in the early acute phase. Furthermore, the elevation of those biomarkers often lags behind in the event of bloodstream infections, whereas a rise in temperature may be a comparatively reliable early sign. Meanwhile, there are other reasons for fever than infection in burn patients [13].

Nearly half of the patients (44%) received at least one transfusion of blood products. Among these, the most frequent product was erythrocyte concentrate (EC) with a median transfused volume of 1785 mL per patient receiving EC. This can be considered a substantial quantity as less than a third of the burn patients being transfused had a severe burn injury (TBSA > 20%). As one unit of erythrocyte concentrate equals 255 mL in our hospital, this also indicates a high transfusion rate in spite of the restrictive transfusion policy with a limit of Hb < 7 g/dL. Although DBL was substantial, it is important to remember that other causes of anaemia and, thereby, the need for blood transfusion, such as blood loss during surgery and dressing changes, were not accounted for in this study.

Findings consistent with the literature on burns included that female patients constituted a minority, were significantly older, and had a different distribution of burn mechanisms than male patients. The fact that flames constitute the most common burn mechanism is not novel [14] and neither is the discovery that blood gases are one of the most frequently ordered tests in intensive care units [15,16,17]. What our study adds to the field, however, is that DBL in burn patients is significant not only in injuries exceeding 20% TBSA but also in minor burns.

Our estimation of DBL resulting in a median of 13.1 mL/day aligns with the results of Chant et al. (mean 13.3 mL/day) who studied a mix of medical and surgical ICU patients with an LOS of at least 30 days [15]. This setting is more comparable to the burn patient cohort used in many other studies on the topic that focus on the acute phase of critical illness, in which more diagnostic sampling is expected and, therefore, higher amounts of DBL/day have been reported. Length of hospital stay is an important parameter in these calculations, which of course influences the endpoint of DBL per day. Longer LOS yields an increased total DBL but a decrease in DBL/day, based on the assumption that diagnostic testing becomes less frequent during hospitalisation. This may explain the negative correlation between burn injury extent and DBL/%TBSA/day displayed in this report since a higher %TBSA generally promotes a longer LOS.

Yao et al. reported a median DBL/day per patient of 6.8 mL/day in patients with severe burns > 40% TBSA [11], which is lower than in our patient cohort with a median of 13.1 mL/day per patient. Perhaps the discrepancy can be explained by the assumption that more severely burnt patients in general have a longer LOS, which means DBL/day will be spread out over more days and hence result in a lower DBL/day, or by the fact that our LOSs were shorter. In our study, taking place in one of two national burn centres in Sweden, LOS was further influenced by the fact that referring hospitals tend to take back their patients as early as possible (i.e., after the acute phase). This means that, theoretically, the patients’ total LOS was longer than the numbers we were working with in this report, since hospitalisation may have continued in their local hospital.

Notably, the assumption that testing becomes less frequent with increasing LOS is supported by the results from the study conducted by Yao et al. in severely burned patients [11] but does not correspond to what was described by Chant et al. in their study where the frequency of diagnostic blood testing was nearly the same for patients with a LOS of more than 40 days as for newly admitted ICU patients [15]. This may be explained by varying reasons for ICU care, differences between patient populations, and local traditions concerning blood-drawing routines.

As the mean value of our endpoint DBL/%TBSA/day was greatly influenced by outliers, we decided to present the median value in our results. Such outliers were, for instance, electric injuries, which yield a small burn area but indeed severe damage, requiring hospital care. Speculating, deviant values could also have appeared if a patient with a smaller burn extent suffered complications such as a severe infection requiring blood cultures, which could lead to a longer LOS and greater blood loss than their %TBSA would have suggested. A logistic regression analysis comparing DBL/%TBSA/day in relation to patient-specific parameters indicated that DBL per %TBSA and day was higher in minor burns—a possible indicator that minor burns were subject to a more deliberate blood sampling routine during a relatively short length of stay as compared to major burns.

Because of the presence of indwelling catheters, from which all blood samples were drawn, it was decided that it was rational to assume that every blood sampling device was filled to nominal capacity when estimating the blood loss. Our observed result of an average discarded volume of 2.5 mL blood per sampling from indwelling catheters in the burn centre was not included in our estimation of diagnostic blood loss (see Section 2), and therefore, our results can be considered lower limits of the amount of blood that was in fact lost.

The retrospective character of the study, resulting in the fact that the amount of DBL from blood draws could only be estimated, remains the strongest limitation of the study. It is likely that DBL was rather under- than overestimated with the methods applied since possible excess blood loss during blood draws was not taken into account. Furthermore, the present data did not allow us to deduce information on at which stage of therapy DBL was highest. Haemoglobin levels were not assessed in our patients, which did not allow us to correlate the risk for HAA with DBL in our patients. Additionally, confounders of anaemia and blood loss from surgical procedures, wound dressing changes, anticoagulants, renal failure, infections, or comorbidities were not assessed.

All adult patients admitted to the burn centre during the studied time period were enrolled in the study. This measure was taken deliberately, as patients with minor burns also frequently received treatment at the BICU instead of the normal ward. Possible bias was ruled out by weighing daily DBL against %TBSA and the Baux score.

## 5. Conclusions

The findings of this study demonstrate that daily blood loss from diagnostic testing in burn patients is greatly influenced by burn injury extent and length of stay. These findings are comparable to those of other patients receiving intensive care for a longer period of time. The total quantity of diagnostic blood loss can reach significant levels and may have an impact on the transfusion rate, which is considerable among these patients. Also, it was endorsed that phlebotomy waste may add up to a substantial amount of blood, which must not be overlooked when assessing iatrogenic blood loss.

Overall, our results can hopefully raise awareness of the substantial volume diagnostic blood loss may add up to and motivate clinicians to think twice when ordering routine blood samplings so that the patients do not become victims of daily routine. It would seem feasible to establish a more restrictive blood sampling protocol and also consider the use of more modern devices that allow for analysis with lower blood volumes.

We believe we have demonstrated one important source of anaemia in burn patients that is under-reported and underappreciated. Further work is required to consolidate our findings, for example, undertaking similar analyses accounting for known causes of anaemia including blood loss from surgical procedures, wound dressing changes, anticoagulants, and sepsis as well as comorbidities. Perhaps the most important of these is renal failure, which can result in prolonged and resistant anaemia. Such analysis will elucidate better compounding factors versus confounding factors.

## Figures and Tables

**Table 1:** Demographics and clinical data: total and divided by gender. * Statistically significant *p*-values.

Parameter	Total	Female	Male	*p*-Value
n (%)	166 (100)	56 (33.7)	110 (66.3)	-
Age (years), mean (±SD)	53.2 (±20.1)	57.6 (±21.1)	51.0 (±19.3)	0.046 *
LOS (days), mean (±SD)	17.0 (±40.7)	17.0 (±40.3)	17.1 (±41.0)	0.992
Aetiology of burn, n (%)				
Flame	127 (76.5)	38 (67.9)	89 (80.9)	0.061
Scald	22 (13.3)	15 (26.8)	7 (6.4)	<0.001 *
Electric	12 (7.2)	1 (1.8)	11 (10.0)	0.053
Contact	3 (1.8)	1 (1.8)	2 (1.8)	0.988
Chemical	2 (1.2)	1 (1.8)	1 (0.9)	0.625
%TBSA, mean (±SD)	18.0 (±20.1)	16.0 (±18.3)	19.0 (±20.9)	0.368
n needing surgery (%)	124 (74.7)	40 (71.4)	84 (76.4)	0.489
n of operations, mean (±SD)	1.9 (±3.1)	1.7 (±2.9)	2.1 (±3.1)	0.422
n needing dialysis (%)	6 (3.6)	2 (3.6)	4 (3.6)	0.983
n of days with CRRT, mean (±SD)	0.6 (±7.1)	0.1 (±0.7)	0.9 (±8.8)	0.506
n needing mechanical ventilation (%)	42 (25.3)	12 (21.4)	30 (27.3)	0.413
n of days in respirator, mean (±SD)	1.4 (±3.6)	1.2 (±4.0)	1.5 (±3.5)	0.665
Mortality, n (%)	20 (12.0)	7 (12.5)	13 (11.8)	0.898
Baux score, mean (±SD)	71.2 (±27.0)	73.6 (±29.4)	70.0 (±25.8)	0.419
LOS per %TBSA, mean (±SD)	1.9 (±4.0)	2.2 (±3.6)	1.7 (±4.2)	0.538

**Table 2:** Volume and content for each blood test category.

Category	Volume (mL)	Content
Blood culture	10	
Blood gas	2	pH, pCO_2_, pO_2_, O_2_sat, HCO_3_, BE, lactate, Na, K, iCa^2+^ activity, Cl^−^, EVF, Hb, glucose
CRRT post-filter ionised calcium (CRRT iCa^2+^)	2	
Complete blood count (CBC)	4	
Coagulation	3.5	APTT, fibrin, d-dimer, fibrinogen, PT (INR), antithrombin
Chemistry	5	CRP, ALAT, ASAT, ALP, bilirubin, bilirubin conjugated, calcium, GFR, phosphate, creatinine, magnesium, urea, sodium, potassium, LD, albumin, CKMB, CK, troponin I, myoglobin, triglycerides
Other	5	S-Osmolality, S-CDT

Notes: BE = base excess, EVF = erythrocyte volume fraction, CRRT = continuous renal replacement therapy, APTT = activated partial thromboplastin time, PT (INR) = prothrombin international normalised ratio, CDT = carbohydrate-deficient transferrin.

**Table 3:** Blood tests and mean estimated blood loss in patients in which the respective test was performed.

Test Category	Performed in n of Patients (%)	Number of Tests	Mean Estimated Blood Loss per Patient in mL (±SD)
Total	166 (100)	15,397	333.5 (±864.2)
CBC	159 (95.8)	2006	50.5 (±118.3)
Chemistry	158 (95.2)	1980	62.7 (±146.7)
Coagulation	155 (93.4)	1363	30.8 (±72.6)
Other	146 (88.0)	428	14.7 (±28.8)
Blood gas	115 (69.3)	8083	140.6 (±298.2)
Blood culture	80 (48.2)	1411	176.4 (±393.6)
CRRT iCa^2+^	9 (5.4)	126	28.0 (±24.6)

Notes: CBC = complete blood count. CRRT iCa^2+^ = continuous renal replacement therapy ionised calcium.

**Table 4:** Median diagnostic blood loss per patient in relation to burn extent.

%TBSA	Number of Patients	Median Total DBL/Patient in mL (IQR)	Median DBL/d/Patient in mL (IQR)
0–20.0	117	48.5 (27.8, 134.3)	10.1 (6.2, 17.5)
20.1–50.0	36	523.5 (258.4, 1026.5)	26.2 (16.6, 39.2)
50.1–100	13	152.0 (36.3, 1683.0)	36.5 (23.3, 62.2)
**Parameter**		**Median volume in mL, (IQR)**	
Total DBL/patient		87.5 (34.6, 304.8)	
DBL/day/patient		13.1 (7.0, 23.9)	
DBL/%TBSA/patient		10.8 (4.6, 27.8)	
DBL/%TBSA/day/patient		1.2 (0.7, 2.3)	

**Table 5:** Demographic and clinical data: total and divided by low and high DBL/d. * Statistically significant *p*-values.

Parameter	Total	Low DBL/day (<13.1 mL/d)	High DBL/day (≥13.1 mL/d)	*p*-Value
n (%)	166 (100)	56 (33.7)	110 (66.3)	-
Age (years), mean (±SD)	53.2 (±20.1)	53.7 (±21.2)	52.7 (±18.9)	0.759
Female gender, n (%)	56 (33.7)	33 (38.4)	23 (28.8)	0.190
LOS (days), mean (±SD)	17.0 (±40.7)	10.1 (±9.7)	24.5 (±56.9)	0.029 *
Aetiology of burn, n (%)				
Flame	127 (76.5)	60 (69.8)	67 (83.8)	0.034 *
Scald	22 (13.3)	15 (17.4)	7 (8.8)	0.099
Electric	12 (7.2)	6 (7.0)	6 (7.5)	0.897
Contact	3 (1.8)	3 (3.5)	0 (0)	0.092
Chemical	2 (1.2)	2 (2.3)	0 (0)	0.170
%TBSA, mean (±SD)	18.0 (±20.1)	10.3 (±12.0)	26.2 (±23.5)	<0.001 *
n needing surgery (%)	124 (74.7)	59 (68.6)	65 (61.3)	0.061
n of operations, mean (±SD)	1.9 (±3.1)	1.1 (±1.0)	2.9 (±4.1)	<0.001 *
n needing dialysis (%)	6 (3.6)	1 (1.2)	5 (6.3)	0.079
n of days with CRRT, mean (±SD)	0.6 (±7.1)	0.0 (±0.3)	1.3 (±10.3)	0.275
n needing mechanical ventilation (%)	42 (25.3)	8 (9.3)	34 (42.5)	<0.001 *
n of days in respirator, mean (±SD)	1.4 (±3.6)	0.3 (±0.9)	2.6 (±4.9)	<0.001 *
Mortality, n (%)	20 (12.0)	6 (7.0)	14 (17.5)	0.037 *
Baux score, mean (±SD)	71.2 (±27.0)	64.0 (±23.5)	79.0 (±28.5)	<0.001 *
LOS per %TBSA, mean (±SD)	1.9 (±4.0)	2.2 (±3.4)	1.6 (±4.5)	0.290

## Data Availability

The authors are willing to make their data, analytic methods, and study materials available to other researchers upon reasonable request, including relevant ethical and legal permissions to the corresponding author.
